# Effect of S-PRG Eluate on Biofilm Formation and Enzyme Activity of Oral Bacteria

**DOI:** 10.1155/2012/814913

**Published:** 2012-05-27

**Authors:** Masahiro Yoneda, Nao Suzuki, Yosuke Masuo, Akie Fujimoto, Kosaku Iha, Kazuhiko Yamada, Tomoyuki Iwamoto, Takao Hirofuji

**Affiliations:** ^1^Center for Oral Diseases, Fukuoka Dental College, 3-2-1 Hakataekimae, Hakata-ku 812-0011, Japan; ^2^Section of General Dentistry, Department of General Dentistry, Fukuoka Dental College, 2-15-1 Tamura, Sawara-ku, Fukuoka 814-0193, Japan

## Abstract

Recently, the antibacterial activity of a composite resin containing prereacted glass ionomer (S-PRG) filler was revealed. We examined the effect of an S-PRG eluate on various biologic activities of *Streptococcus mutans* and *Porphyromonas gingivalis*. Adherence ability of *S. mutans* was evaluated by microtiter plate assay; protease and gelatinase activities of *P. gingivalis* were examined by synthetic substrate hydrolysis and gelatin film spot assay, respectively. Coaggregation of *P. gingivalis* with *Fusobacterium nucleatum* was also examined. S-PRG eluate was found to suppress streptococcal adherence. S-PRG eluate inhibited the protease and gelatinase activities of *P. gingivalis* and the coaggregation between *P. gingivalis* and *F. nucleatum*. These results indicate that S-PRG eluate suppresses streptococcal adherence and inhibits the protease and coaggregation activities of *P. gingivalis*. These findings may prompt research into novel strategies for preventing caries and periodontitis.

## 1. Introduction

Dental caries and periodontitis are two major causes of tooth loss in adults. Dental caries arises from the interplay between the oral flora, teeth, and dietary factors. Among the most important cariogenic bacteria is *Streptococcus mutans*, which adheres tenaciously to glucan-coated surfaces, produces large amounts of extracellular polysaccharides, and is highly acidogenic and acid-tolerant [1–4]. The colonization of dental plaque by *S. mutans *plays a causative role in dental caries. Sugar metabolism is central to the behavior of mutans streptococci, and sucrose, the most cariogenic dietary carbohydrate, is used to produce the extracellular polysaccharides that form the biofilm matrix, which facilitates the association of mutans streptococci with dental plaque.

 Periodontitis is caused by periodontopathic bacteria such as *Porphyromonas gingivalis, *a black-pigmented, Gram-negative, asaccharolytic anaerobic bacterium [5]. *P. gingivalis* has several biologic activities such as protease secretion and coaggregation [6, 7]. We have previously isolated the protease gingipain and reported its suppressive activity on human neutrophils [8] and its virulence in a mouse abscess model [9]. Gingipain is related to the growth promotion of *P. gingivalis* by *Tannerella forsythensis* [10]. Gingipain is also associated with gelatinase activity [11], which may cause periodontal tissue degradation. *P. gingivalis* coaggregates with other oral bacteria such as *Fusobacterium nucleatum*, and the formation of these multistrain complex communities is an initial and critical step in the pathogenesis of periodontitis [12].

Suppression of bacterial activity is considered a key modality in controlling dental caries and periodontal diseases. Various strategies have been developed, including inhibition of protease activity [13, 14] and the application of antibacterial dental materials [15, 16]. Recently, the antibacterial activity of a composite resin containing prereacted glass ionomer (S-PRG) filler was revealed [17]. The S-PRG filler particles are formed by an acid-base reaction between fluoroaluminosilicate glass and polyacrylic acid [18] and are capable of fluoride release and recharge [19, 20]. Here, we report that an S-PRG eluate has a suppressive effect on adherence of *S. mutans* and on the proteolytic, gelatinase, and coaggregation activity of *P. gingivalis*.

## 2. Methods and Materials

### 2.1. Bacterial Strains and Culture Conditions

*Streptococcus mutans* JCM 5705 were cultured in GAM broth (Nissui Pharmaceutical Co., Ltd., Tokyo, Japan) with 2.0% sucrose for 24 h.* P. gingivalis *ATCC 33277 were maintained on CDC anaerobic blood agar (Becton Dickinson, Cockeysville, MD, USA) in an anaerobic atmosphere (80% N_2_, 10% H_2_, 10% CO_2_) and inoculated into tryptic soy broth (Difco Laboratories, Detroit, MI, USA) supplemented with hemin (5 *μ*g/mL) and menadione (1 *μ*g/mL). *F. nucleatum *ATCC 10953 and 25585 were grown as previously reported [21].

### 2.2. Human Saliva Preparation

Whole saliva samples stimulated by the chewing of paraffin gum were collected from three healthy human participants (mean age: 31.7 ± 5.5 years). The mixed saliva was centrifuged at 10,000 *×*g for 20 min at 4°C, and the supernatant was incubated at 56°C for 30 min to inactivate complements, which may affect the viability of bacteria [22]. The samples were sterilized using a sterile membrane filter (pore size: 0.22 *μ*m; Millipore, Billerica, MA, USA) and used immediately for the bacterial adherence assay.

### 2.3. Bacterial Adherence Assay in 96-Well Microtiter Plates

Bacterial adherence by each strain was assayed using a previously described method [23]. To start the bacterial adherence assay, precultures of *S. mutans* stored at −80°C were grown in 10 mL of brain-heart infusion (BHI) medium (Difco Laboratories, Detroit, MI, USA) for 24 h at 37°C to full growth. To evaluate bacterial adherence, 20 *μ*L of bacterial cell suspension and 180 *μ*L of tryptic soy broth (without dextrose, supplemented with 0.25% sucrose; TSBS; Wako Pure Chemical Industries, Osaka, Japan) were added to each well of a flat-bottomed 96-well microtiter plate (Nunc A/S, Roskilde, Denmark), which was precoated with human saliva. To evaluate the effect of S-PRG eluate on bacterial adherence, different percentages of S-PRG were substituted for the culture medium. For the control, phosphate-buffered saline (PBS) was used instead of S-PRG. The plates were then incubated at 37°C for 16 h under anaerobic conditions, and the liquid medium was removed. The wells were rinsed a second time with distilled water, air-dried, and stained with 0.25% safranin for 15 min. After staining, the plates were rinsed with distilled water to remove excess dye and then air-dried. The bacterial mass was dissolved with ethanol, and the stained solubilized bacterial matter in each sample was quantified by measuring absorbance at 492 nm using a microplate reader (Sunrise Rainbow Thermo; Tecan Group, Männedorf, Switzerland).

### 2.4. Preparation of Sonicated Extracts of *P. gingivalis* Cells

*P. gingivalis* cells of late logarithmic stage in tryptic soy broth were harvested by centrifugation and washed with PBS. Fifty micrograms of these bacteria were suspended in 0.5 mL of PBS, and the cells were disrupted by sonication on ice [24]. Intact cells were removed by centrifugation and the protein concentration of the supernatant was measured.

### 2.5. Preparation of S-PRG Eluate

S-PRG eluate was prepared by the method of Fujimoto et al. [25], and the condition, which produces the high concentration of each ion, was applied. Briefly, S-PRG filler (SHOFU Inc., Kyoto, Japan) was mixed with an equal amount of distilled water and shaken gently at room temperature for 24 h. Filler material was removed by filtration. The ion solution was centrifuged to remove any residual insoluble material, and the clear supernatant collected was used as the S-PRG eluate. Elemental analysis of ions (Al, B, Na, Si, and Sr) released from S-PRG filler was performed using inductively coupled plasma atomic emission spectroscopy (ICP-AES; ICPS-8000, Shimadzu Co., Kyoto Japan). Analysis was conducted after preparing calibration curves corresponding to each element (standard solution concentration; Si: 0, 0.5, 1, and 5 ppm; Sr: 0, 5, 20, and 50 ppm; B: 0, 10, 50, and 100 ppm; Al: 0, 0.5, 5, and 10 ppm; Na: 0, 0.5, 20, and 50 ppm). Concentration of F was also determined using a fluoride ion electrode method after preparing its calibration curves (standard solution concentration: 0.1, 1, 5, and 10 ppm). A fluoride electrode (Model 9609BN, Orion Research Inc., MA, USA) connected to a pH/ion meter (720A, Orion Research Inc.) was used to measure the F concentration of each solution. Each test solution was diluted to obtain a fluoride ion level of less than 5 ppm, and 0.5 mL of an ionic strength adjuster (TISAB III, Orion Research Inc.) was added to 5 mL of each diluted solution to measure F ion concentration. For each ion under investigation, the amount released into the solution was expressed in ppm, as well as a cumulative amount in per gram of S-PRG filler weight (mg/g). To exclude the effect of ions in saliva preparation, no saliva was added to the reaction mixtures of each experiment.

### 2.6. Protease Activity of *P.-gingivalis*-Sonicated Extract

N_*α*_-benzoyl-L-arginine 4-nitroanilide hydrochloride (BAPNA) was purchased from Sigma Aldrich (St. Louis, MS, USA). BAPNA is a synthetic substrate, which develops yellow color when the site of arginine is digested by trypsin-like proteases. Enzyme assay was performed according to the method of Potempa et al. [26]. Briefly, *P.-gingivalis-*sonicated extract was added to an aqueous reaction mixture containing 1 mM BAPNA, 0.2 M Tris-HCl (pH 7.5), 0.1 M NaCl, 5 mM CaCl_2_, and 10 mM cysteine. For the control, *P.-gingivalis-*sonicated extract was removed from the reaction mixture. The background color is calculated by measuring the optical density of *P.-gingivalis-*sonicated extract. To analyze the effect of S-PRG, the distilled water in the reaction mixture was replaced with S-PRG solution. The enzyme assay mixture was incubated at 37°C for 30 min and a colorimetric assay was performed (OD 405 nm) to determine colored metabolites (as a measure of protease activity).

### 2.7. Gelatinase Activity of *P.-gingivalis*-Sonicated Extract

A gelatinase spot assay was performed using a modification of the method of Sumantran et al. [27]. Different concentrations of S-PRG eluate were added to the reaction mixture containing 1 mM BAPNA, 0.2 M Tris-HCl (pH 7.5), 0.1 M NaCl, 5 mM CaCl_2_, and 10 mM cysteine. Different concentrations of *P.-gingivalis-*sonicated extract were also added and the mixture was serially diluted with distilled water. A 15 *μ*L spot of each mixture was placed onto gelatin-coated X-ray film (Kodak Ultraspeed, Eastman Kodak Company, Rochester, NY, USA) and incubated at 37°C in a 100% humidified atmosphere. For the control, *P. gingivalis *SE was removed from the reaction mixture. After 2 h, the X-ray film was removed from the humidified box and washed with tap water. In gelatinase-positive samples, the surface of the film in the application area was removed, revealing the base color of the film.

### 2.8. Coaggregation between *P. gingivalis* and *F. nucleatum*

A coaggregation assay was performed according to the method of Kinder and Holt [28]. Briefly, bacterial cells were harvested and washed twice by centrifugation (10,000 ×g for 15 min at 4°C) and then resuspended in prereduced coaggregation buffer (CB; 10 mM Tris, 0.15 M NaCl, 0.1 mM CaCl_2_, 0.1 mM MgCl_2_·6H_2_O (pH 7.5)) to an optical density of 2.0 at 660 nm. Strains to be examined for coaggregation were combined with an equal volume of a test strain (or CB as a control) in a total volume of 2 mL and incubated anaerobically. To evaluate the effect of S-PRG eluate, 10%, 50%, or 100% of the distilled water in the CB was replaced with S-PRG eluate. After 30 and 60 min, the reaction mixtures were evaluated by a visual scoring system (0, +1, +2, +3, +4) described by Kinder and Holt [28]. All assays were repeated at least three times. Representative results are shown, with an indication of reproducibility.

## 3. Results

### 3.1. Nature of S-PRG Eluate

The result of ICP-AES and fluoride ion electrode method showed that the ion concentration of S-PRG eluate was as follows: Al, 40.8 ppm; B, 1907.8 ppm; Si, 28.1pm; Sr, 218.3 ppm; and F, 127.0 ppm. The pH of the eluate was 7.8 and all the experiments were performed with this single batch. No pH change and precipitation occurred when the eluate was added to the reaction mixtures.

### 3.2. Effect of S-PRG Eluate on Streptococcal Adherence

**Figure 1:**
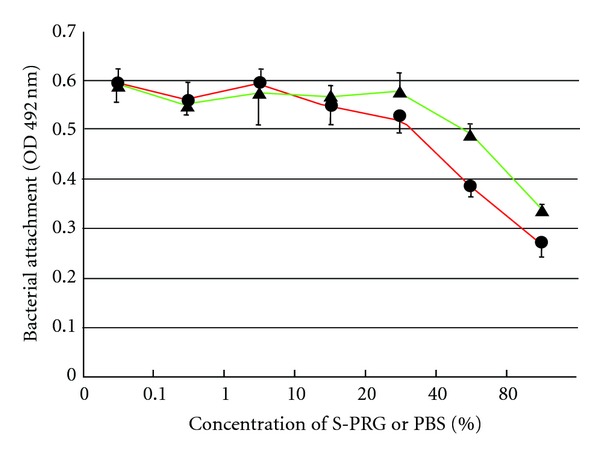
Adherence of *Streptococcus mutans* JCM 5705 in the presence and absence of PBS or S-PRG eluate. To evaluate the effect of S-PRG eluate on adherence, different percentages of S-PRG were substituted for culture medium (circle). For the control, phosphate-buffered saline was used instead of S-PRG (triangle). The plates were incubated at 37°C for 16 h under anaerobic conditions, stained with 0.25% safranin, and the amount of stain in an ethanol-solubilized sample quantified by measuring absorbance at 492 nm. Data are representative of three independent experiments and the bars indicate the standard deviation.

Substitution of culture medium with either PBS or S-PRG reduced adherence by *S. mutans*. S-PRG showed the greatest suppressive effect (Figure 1). When more than 20% of culture medium was substituted for S-PRG eluate, approximately 20% less bacteria were adhered.

### 3.3. Effect of S-PRG on the Protease Activity of *P. gingivalis*

**Figure 2:**
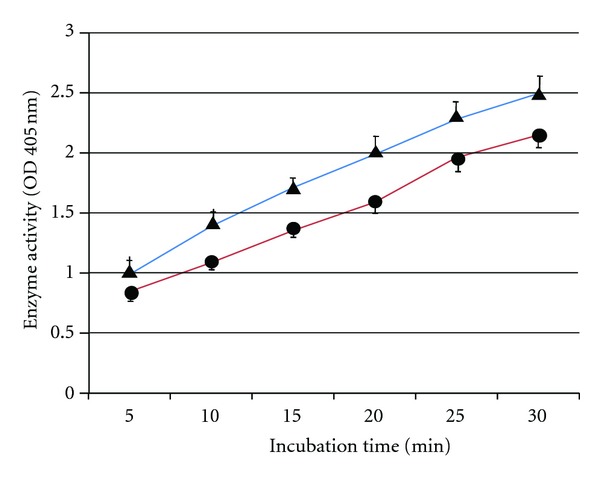
BAPNA hydrolysis activity (as an indicator of protease activity) in the presence and absence of S-PRG eluate.* P.-gingivalis-*sonicated extract was added to the reaction mixture. To analyze the effect of S-PRG, distilled water in control mixture (triangle) was replaced with S-PRG solution (circle). Colored metabolites were quantified using a colorimetric absorbance assay at 405 nm. Data are representative of three independent experiments and the bars indicate the standard deviation.

A sonicated extract sample, prepared from 50 *μ*g/mL *P. gingivalis* cells, was used. S-PRG showed a 20% inhibitory effect on protease activity (Figure 2). However, no effect of S-PRG was observed when the amount of *P.-gingivalis*-sonicated extract was increased or decreased.

### 3.4. Effect of S-PRG on the Gelatinase Activity of *P. gingivalis*

**Figure 3:**
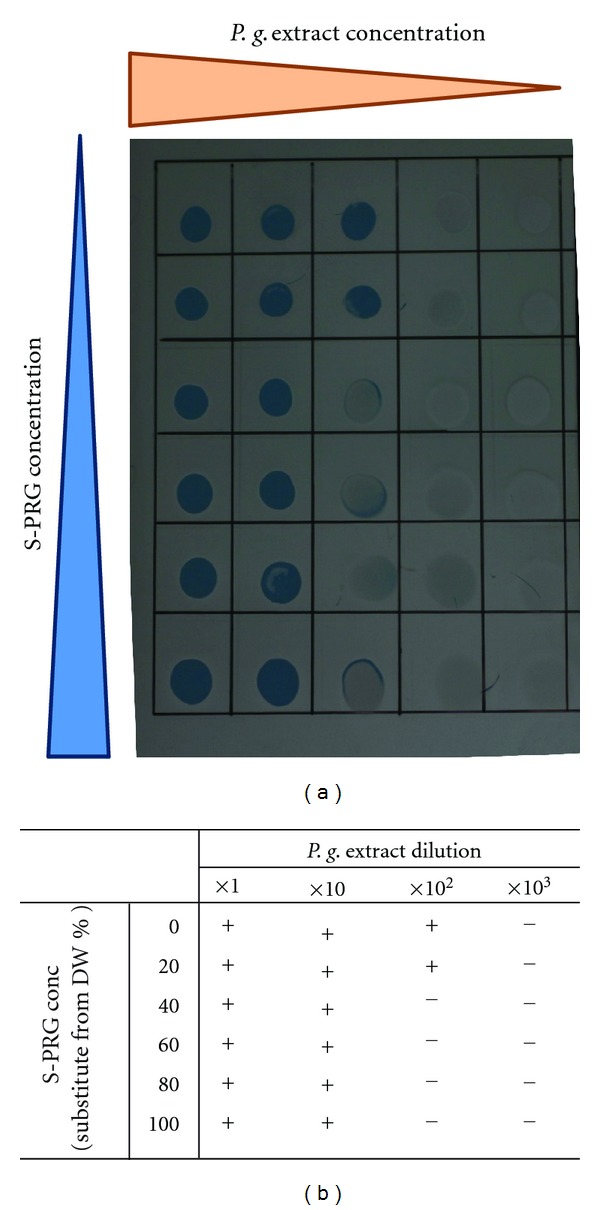
Gelatinase spot assay. Different amounts of S-PRG eluate were added to the reaction mixture. *P.-gingivalis-*sonicated extract was then added and the mixture was serially diluted with distilled water. A 15 *μ*L spot of each mixture was placed onto gelatin-coated X-ray film and incubated at 37°C for 2 h in a humidified atmosphere before being washed with tap water. Removal of the gelatin surface beneath the spotted area revealed the base color of the film and indicated the presence of gelatinase activity. (a) Photograph of X-ray film after assay. (b) Results of gelatinase spot assay.

Figure 3 shows the result of the gelatinase spot assay. S-PRG inhibited the gelatinase activity of *P. gingivalis*. This inhibitory effect was observed at a 100-fold dilution of sonicated extract. S-PRG eluate and the buffer did not show any effect on the gelatin film.

### 3.5. Effect of S-PRG on the Coaggregation between *P. gingivalis* and *F. nucleatum*

**Figure 4:**
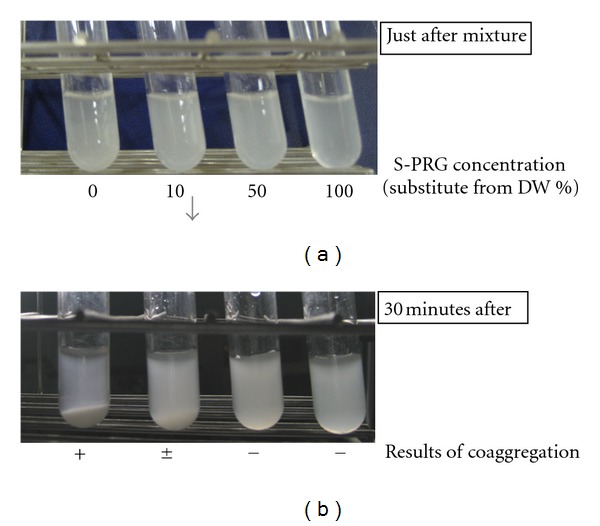
Coaggregation between *P. gingivalis* and *F. nucleatum* in the presence and absence of S-PRG eluate. (a): photographs of coaggregation. (b): results of coaggregation assay.

*F. nucleatum *ATCC25585 exhibited coaggregation with *P. gingivalis* ATCC33277. Conversely, *F. nucleatum *ATCC10953 did not coaggregate with *P. gingivalis *ATCC33277. S-PRG inhibited coaggregation, as shown in Figure 4. When 10% of CB was substituted for S-PRG eluate, only weak coaggregation was observed between *P. gingivalis* and *F. nucleatum *ATCC25585. When 50% of CB was substituted for S-PRG eluate, no coaggregation was observed between these strains.

## 4. Discussion

S-PRG fillers are widely used in many fields of dentistry, such as composite resins, cements, dentures, sealants, and so on [29], partly because of their ability to recharge with fluoride ions, the subsequent release of which promotes dentin remineralization. Therefore, the role of S-PRG in preventing caries formation has been investigated.

As we have reported here, S-PRG eluate inhibits adherence by *S. mutans*. The first step of bacterial infection is adherence to the host, and inhibition of this bacterial adherence may become one of the effective means of preventing dental caries. Recently, an *in vivo* experiment showed that less dental plaque was formed on an S-PRG-containing resin block than on two alternative materials [17]. In addition, the adherence of *S. mutans* to the saliva-treated resin surface was significantly lower on the S-PRG-containing resin than that on the other two materials, despite none of the materials possessing significant bactericidal activity [17]. From these results, we can conclude that S-PRG can inhibit *S. mutans* in both solid resin and soluble forms.

S-PRG eluate was found to have a suppressive effect on the BAPNA-hydrolyzing and gelatinase activities of *P. gingivalis*. The proteolytic activity of *P. gingivalis* is derived mainly from its production of gingipains. These are known to be associated with tissue destruction and host immune disturbance, which results in the progression of periodontitis, so suppression of gingipain activity should be advantageous in maintaining periodontal health. Gelatinase is related to progression of secondary caries underneath tooth restorations [30]. Santos et al. reported that zinc oxide cement and amalgam suppress gelatinase activity, which may contribute to the caries preventative effects of these materials [31]. It is already known that S-PRG limits caries progression due to its release of fluoride [25, 32], but we find that it may additionally prevent secondary caries by inhibiting gelatinase activity at restoration sites. The gelatinase spot assay, which was performed in this work, is not very sensitive and we could only determine whether it is positive or negative. We need to perform more specific assay to clarify the effect of S-PRG on gelatinase activity.

S-PRG had a suppressive effect on the coaggregation between *P. gingivalis* and *F. nucleatum*. Coaggregation of periodontopathic bacteria is associated with bacterial attachment in the gingival crevice [33]. Recently, some metal ions were found to suppress the coaggregating ability of *P. gingivalis* and they are expected to inhibit the settlement of *P. gingivalis* in the gingival sulcus [34]. S-PRG may also disturb the formation of advanced multistrain bacterial communities in the periodontal environment.

The mechanism of these inhibitory effects is unclear and needs to be clarified. S-PRG is known to release various ions, including F^−^, Al^3+^, Sr^2+^, SiO^3−^, BO_3_^3−^, and Na^+^ [25, 35]. Boron is known to have an antibacterial activity in cutaneous diseases and periodontitis [36, 37] and inhibits bacterial and fungal quorum sensing [38]. Quorum sensing is a key factor in biofilm formation, so inhibition of this function in Streptococci may be a good candidate mechanism underlying the actions of S-PRG. In *P. gingivalis*, the mechanism responsible for S-PRG actions may involve the control of metal salts and ions that regulate bacterial enzyme activity. Gingipains are known to require metal ions to achieve maximum enzyme activity [39], whereas gelatinases are inhibited by metal salts [40]. Thus, S-PRG may affect enzyme activity by modulating the concentrations of these metal salts and ions.

Some experiments in this report were not suitable for statistical analysis. We instead repeated each assay to confirm its reproducibility and have presented representative data. Further work is now in progress to obtain more statistical data and characterize the inhibitory mechanism of S-PRG eluate on *P. gingivalis*. Saku et al. performed both *in vitro* and *in vivo* experiments [17]. Here we reported *in vitro* data, and we need to further perform *in vivo* experiments to confirm the biological effect of S-PRG.

In summary, this is the first report showing the effect of an S-PRG eluate on streptococcal adherence and on the protease and coaggregation activities of *P. gingivalis*. These findings may prompt research into novel strategies for preventing caries and periodontitis.

## References

[1] Hardie JM, Whiley RA, Newman HN, Wilson M (1999). Plaque microbiology of crown caries. *Dental Plaque Revisited Cardiff*.

[2] Beighton D, Brailsfold SR, Newman HN, Wilson M (1999). Plaque microbiology of crown caries. *Dental Plaque Revisited Cardiff*.

[3] Hamada S, Slade HD (1980). Biology, immunology, and cariogenicity of *Streptococcus mutans*. *Microbiological Reviews*.

[4] Loesche WJ (1986). Role of *Streptococcus mutans* in human dental decay. *Microbiological Reviews*.

[5] Genco CA, Potempa J, Mikolajczyk-Pawlinska J, Travis J (1999). Role of gingipains R in the pathogenesis of *Porphyromonas gingivalis*-mediated periodontal disease. *Clinical Infectious Diseases*.

[6] Kadowaki T, Nakayama K, Okamoto K (2000). *Porphyromonas gingivalis* proteinases as virulence determinants in progression of periodontal diseases. *Journal of Biochemistry*.

[7] Kolenbrander PE, Andersen RN (1989). Inhibition of coaggregation between *Fusobacterium nucleatum* and *Porphyromonas (Bacteroides) gingivalis* by lactose and related sugars. *Infection and Immunity*.

[8] Kadowaki T, Yoneda M, Okamoto K, Maeda K, Yamamoto K (1994). Purification and characterization of a novel arginine-specific cysteine proteinase (argingipain) involved in the pathogenesis of periodontal disease from the culture supernatant of *Porphyromonas gingivalis*. *Journal of Biological Chemistry*.

[9] Yoneda M, Hirofuji T, Anan H (2001). Mixed infection of *Porphyromonas gingivalis* and *Bacteroides forsythus* in a murine abscess model: involvement of gingipains in a synergistic effect. *Journal of Periodontal Research*.

[10] Yoneda M, Yoshikane T, Motooka N (2005). Stimulation of growth of *Porphyromonas gingivalis* by cell extracts from *Tannerella forsythia*. *Journal of Periodontal Research*.

[11] Andrian E, Mostefaoui Y, Rouabhia M, Grenier D (2007). Regulation of matrix metalloproteinases and tissue inhibitors of matrix metalloproteinases by *Porphyromonas gingivalis* in an engineered human oral mucosa model. *Journal of Cellular Physiology*.

[12] Kamaguchi A, Nakayama K, Ohyama T, Watanabe T, Okamoto M, Baba H (2001). Coaggregation of *Porphyromonas gingivalis* and *Prevotella intermedia*. *Microbiology and Immunology*.

[13] Kadowaki T, Yamamoto K (2003). Suppression of virulence of *Porphyromonas gingivalis* by potent inhibitors specific for gingipains. *Current Protein and Peptide Science*.

[14] Inaba H, Tagashira M, Kanda T, Amano A (2011). Proliferation of smooth muscle cells stimulated by *Porphyromonas gingivalis* is inhibited by apple polyphenol. *Journal of Periodontology*.

[15] Cui C, Zhou XN, Chen WM (2011). Self-etching adhesives: possible new pulp capping agents to vital pulp therapy. *Front Medicine*.

[16] Bumgardner JD, Adatrow P, Haggard WO, Norowski PA (2010). Emerging antibacterial biomaterial strategies for the prevention of peri-implant inflammatory diseases. *International Journal of Oral Maxillofacial Implants*.

[17] Saku S, Kotake H, Scougall-Vilchis RJ (2010). Antibacterial activity of composite resin with glass-ionomer filler particles. *Dental Materials Journal*.

[18] Ikemura K, Tay FR, Kouro Y (2003). Optimizing filler content in an adhesive system containing pre-reacted glass-ionomer fillers. *Dental Materials*.

[19] Scougall Vilchis RJ, Yamamoto S, Kitai N, Hotta M, Yamamoto K (2007). Shear bond strength of a new fluoride-releasing orthodontic adhesive. *Dental Materials Journal*.

[20] Han L, Cv E, Li M (2002). Effect of fluoride mouth rinse on fluoride releasing and recharging from aesthetic dental materials. *Dental Materials Journal*.

[21] Yoneda M, Maeda K, Aono M (1990). Suppression of bactericidal activity of human polymorphonuclear leukocytes by *Bacteroides gingivalis*. *Infection and Immunity*.

[22] Jones FS (1927). The effect of heat on antibodies. *Journal of Experimental Medicine*.

[23] Suzuki N, Yoneda M, Hatano Y, Iwamoto T, Masuo Y, Hirofuji T (2011). *Enterococcus faecium* WB2000 inhibits biofilm formation by oral cariogenic streptococci. *International Journal of Dentistry*.

[24] Yoneda M, Hirofuji T, Motooka N (2003). Humoral immune responses to S-layer-like proteins of *Bacteroides forsythus*. *Clinical and Diagnostic Laboratory Immunology*.

[25] Fujimoto Y, Iwasa M, Murayama R, Miyazaki M, Nagafuji A, Nakatsuka T (2010). Detection of ions released from S-PRG fillers and their modulation effect. *Dental Materials Journal*.

[26] Potempa J, Mikolajczyk-Pawlinska J, Brassell D (1998). Comparative properties of two cysteine proteinases (gingipains R), the products of two related but individual genes of *Porphyromonas gingivalis*. *Journal of Biological Chemistry*.

[27] Sumantran VN, Kulkarni AA, Harsulkar A (2007). Hyaluronidase and collagenase inhibitory activities of the herbal formulation *Triphala guggulu*. *Journal of Biosciences*.

[28] Kinder SA, Holt SC (1989). Characterization of coaggregation between *Bacteroides gingivalis* T22 and *Fusobacterium nucleatum* T18. *Infection and Immunity*.

[29] Ikemura K, Tay FR, Endo T, Pashley DH (2008). A review of chemical-approach and ultramorphological studies on the development of fluoride-releasing dental adhesives comprising new pre-reacted glass ionomer (PRG) fillers. *Dental Materials Journal*.

[30] Tjäderhane L, Larjava H, Sorsa T, Uitto VJ, Larmas M, Salo T (1998). The activation and function of host matrix metalloproteinases in dentin matrix breakdown in caries lesions. *Journal of Dental Research*.

[31] Santos MCLG, De Souza AP, Gerlach RF, Trevilatto PC, Scarel-Caminaga RM, Line SRP (2004). Inhibition of human pulpal gelatinases (MMP-2 and MMP-9) by zinc oxide cements. *Journal of Oral Rehabilitation*.

[32] Nakamura N, Yamada A, Iwamoto T (2009). Two-year clinical evaluation of flowable composite resin containing pre-reacted glass-ionomer. *Pediatric Dental Journal*.

[33] Okuda T, Kokubu E, Kawana T, Saito A, Okuda K, Ishihara K (2012). Synergy in biofilm formation between Fusobacterium nulceatum and Prevotella species. *Anaerobe*.

[34] Tamura M, Ochiai K (2009). Zinc and copper play a role in coaggregation inhibiting action of *Porphyromonas gingivalis*. *Oral Microbiology and Immunology*.

[35] Shimazu K, Ogata K, Karibe H (2011). Evaluation of the ion-releasing and recharging abilities of a resin-based fissure sealant containing S-PRG filler. *Dental Material Journal*.

[36] Baker SJ, Akama T, Zhang YK (2006). Identification of a novel boron-containing antibacterial agent (AN0128) with anti-inflammatory activity, for the potential treatment of cutaneous diseases. *Bioorganic and Medicinal Chemistry Letters*.

[37] Luan Q, Desta T, Chehab L, Sanders VJ, Plattner J, Graves DT (2008). Inhibition of experimental periodontitis by a topical boron-based antimicrobial. *Journal of Dental Research*.

[38] Dembitsky VM, Qntar AAAI, Srebnik M (2011). Natural and synthetic small boron-containing molecules as potential inhibitors of bacterial and fungal quarum sensing. *Chemical Reviews*.

[39] Chen Z, Potempa J, Polanowski A, Wikstrom M, Travis J (1992). Purification and characterization of a 50-kDa cysteine proteinase (gingipain) from *Porphyromonas gingivalis*. *Journal of Biological Chemistry*.

[40] De Souza AP, Gerlach RF, Line SRP (2000). Inhibition of human gingival gelatinases (MMP-2 and MMP-9) by metal salts. *Dental Materials*.

